# Idebenone increases mitochondrial complex I activity in fibroblasts from LHON patients while producing contradictory effects on respiration

**DOI:** 10.1186/1756-0500-4-557

**Published:** 2011-12-22

**Authors:** Claire Angebault, Naïg Gueguen, Valérie Desquiret-Dumas, Arnaud Chevrollier, Virginie Guillet, Christophe Verny, Julien Cassereau, Marc Ferre, Dan Milea, Patrizia Amati-Bonneau, Dominique Bonneau, Vincent Procaccio, Pascal Reynier, Dominique Loiseau

**Affiliations:** 1 INSERM U771, Angers 49000, France; 2 CNRS 6214, Angers 49000, France; 3 LUNAM Université, Angers 49000, France; 4 Département de Biochimie et Génétique, Centre Hospitalier Universitaire, Angers 49000, France; 5 Département de Neurologie, Centre Hospitalier Universitaire, Angers 49000, France; 6 Département d'Ophtalmologie, Centre Hospitalier Universitaire, Angers 49000, France; 7 Département de Biochimie et Génétique, CHU d'Angers, 49933 Angers, France

## Abstract

**Background:**

Leber's hereditary optic neuropathy (LHON) is caused by mutations in the complex I subunits of the respiratory chain. Although patients have been treated with idebenone since 1992, the efficacy of the drug is still a matter of debate.

**Methods:**

We evaluated the effect of idebenone in fibroblasts from LHON patients using enzymatic and polarographic measurements.

**Results:**

Complex I activity was 42% greater in treated fibroblasts compared to controls (*p *= 0.002). Despite this complex I activity improvement, the effects on mitochondrial respiration were contradictory, leading to impairment in some cases and stimulation in others.

**Conclusion:**

These results indicate that idebenone is able to compensate the complex I deficiency in LHON patient cells with variable effects on respiration, indicating that the patients might not be equally likely to benefit from the treatment.

## Background

Leber's hereditary optic neuropathy (LHON, OMIM #535000), the most frequent mitochondrial DNA-related disorder, is characterized by maternal inheritance and by incomplete penetrance with male predominance [1,2]. The age of onset is highly variable but typically situated between 15 and 30 years. The acute or subacute onset of the optic neuropathy, due to the specific degenerescence of retinal ganglion cells, generally begins in one eye and affects the other one within a few weeks or months. Three main mitochondrial DNA point mutations (m.11778 G > A in the ND4 gene, m.3460 G > A in the ND1 gene, and m.14484 T > C in the ND6 gene) are responsible for the disease in 95% of patients [1,2]. These mutations affect the protein subunits of complex I of the mitochondrial respiratory chain (NADH dehydrogenase). Fibroblasts from patients carrying one of the three mutations have on average 61% residual complex I activity, associated with defective OXPHOS efficiency and impaired ATP synthesis [3].

Idebenone (2,3-dimethoxy-5-methy-6-(10-hydroxy)-decyl-1,4-benzoquinone), an analog of coenzyme Q, facilitates electron transfer along the respiratory chain. The beneficial effects of this molecule remain controversial since some reports have claimed it leads to faster visual recovery [4-6], whereas other authors have found that idebenone treatment did not alter the course of the disease [7]. A recent randomized controlled trial in 85 patients with LHON showed that patients with discordant visual acuities were the most likely to benefit from idebenone treatment [8]. Another recent retrospective clinical study in 103 LHON patients showed that early and prolonged idebenone treatment may improve significantly the frequency of visual recovery [9] but, within the treated group, only a proportion of patients was shown to respond to the treatment. To date, the biological effects of idebenone on pathological models have received little attention, although idebenone is often administered in cases of LHON. In order to better understand the biological bases of these variable impacts of idebenone in LHON patients, we tested the effects of idebenone on complex I activity and mitochondrial respiration in fibroblasts from patients harbouring one of the three main mutations associated with the disease.

## Methods

### Ethical approval

This work was approved by the Ethical Committee of the University Hospital of Angers (N°2011/39).

### Patients

Fibroblasts were cultured from skin biopsies taken after obtaining informed consent from nine patients (eight men and one woman), each carrying one of the three mutations associated with LHON, and three controls. The LHON mutations and haplotyping were determined in our diagnostics laboratory. The heteroplasmic levels were measured at the same cellular passage numbers as those used for the experiments (Table 1).

**Table 1:** Mitochondrial genetic profile of the LHON fibroblasts

PATIENTS	Age (years)	Sex	mtDNA mutation	Heteroplasmy level	Haplogroup
1	22	M	m.3460G > A	100%	J

2	30	M	m.11778G > A	74%	V

3^a^	43	F	m.11778G > A	100%	U

4	20	M	m.11778G > A	> 90%	K

5	47	M	m.11778G > A	100%	H

6	32	M	m.11778G > A	100%	V

7	44	M	m.11778G > A	81%	J

8	52	M	m.11778G > A	100%	H

9	43	M	m.14484T > C	100%	H

^a^Although Patient 3 had a typical LHON clinical presentation, she was also known to be carrying an *OPA1 *gene mutation (p.Ile382Met). However, the pathogenicity of this mutation has not been fully demonstrated and its contribution to the clinical expression of LHON in this patient is unknown.

### Cell culture

The cells were cultivated in 2/3 Dulbecco's minimum essential medium (Gibco, Cergy-Pontoise, France) supplemented with 1/3 Amniomax (Gibco, Cergy-Pontoise, France) and 10% foetal calf serum (PAA, Pasching, Austria). All experiments were conducted on cells at less than 20 passages to avoid bias due to cell senescence. Ethanol (used as vehicle) or 10 μM idebenone (Santhera Pharmaceutical, Liestal, Switzerland) was added 24 h before each experiment. At this concentration, idebenone has been found to be effective as an antioxidant [10] and capable of stimulating the mitochondrial respiratory chain [11]. Unfortunately, we were not able to perform oxygen consumption in patients 7, 8 and 9.

### Enzymatic activities

The activity of the mitochondrial respiratory chain complexes was measured on cell homogenates in a cell buffer (250 mM saccharose, 20 mM tris[hydroxymethyl]aminomethane, 2 mM EGTA, 1 mg/mL bovine serum albumin, pH 7.2), at 37°C using a Beckman DU 640 spectrophotometer (Beckman Coulter, Brea, CA, USA). The cellular protein content was determined with the Bicinchoninic assay kit (Uptima, Interchim, Montluçon, France) using bovine serum albumin as standard. Complex I (NADH ubiquinone reductase, EC 1.6.5.3) activity was measured according to a procedure described elsewhere [12] and adapted using DCPIP to avoid the inhibition of complex I activity by decylubiquinol [13]. Cells were disrupted by two freezing-thawing cycles, washed, centrifuged for one minute at 16 000 g, re-suspended in the cell buffer (50 μL/10^6 ^cells), and sonicated (6 × 5 s) on ice. Complex I activity was immediately assayed on cell lysate (0.25 × 10^6 ^cells) in the KH_2_PO_4 _buffer (80 mM, pH 7.4), containing 1 mM KCN, 2 mM NaN_3_, 0.075 mM DCPIP and 0.1 mM of decylubiquinone. After 2 min of incubation, 0.3 mM NADH was added and the rate of disappearance of DCPIP was monitored at 600 nm. Rotenone (5 μM) was added during the measurement to determine the background rate, and the activity was calculated using εDCPIP = 19.1 mM^-1 ^cm^-1^. Specific rotenone-sensitive complex I activity was expressed in mIU (nanomoles of DCPIP/min/mg protein). Citrate synthase (EC 2.3.3.1) activity was assayed by a standard procedure [14]. Specific enzymatic activities were expressed in mIU, i.e. nanomoles of 5-5'-dithiobis (2-nitrobenzoic acid), DTNB/min/mg protein. All experiments were performed in triplicate on independent cell cultures.

### Oxygen consumption

The respiratory rates were measured on cells permeabilized by incubation for 2 min with digitonin (15 μg/million cells) and re-suspended in the respiratory buffer (pH 7.4, 10 mM KH_2_PO_4_, 300 mM mannitol, 10 mM KCl, and 5 mM MgCl_2_). The respiratory rates of 3-5 × 10^6 ^cells were recorded at 37°C in 2-ml glass chambers using a high-resolution Oxygraph respirometer (Oroboros, Innsbruck, Austria). Respiration was started with complex I-dependent substrates (5 mM malate/5 mM pyruvate). Complex I-coupled state 3 respiration was measured by adding 0.5 mM NAD^+^/1.5 mM ADP. Then, 10 mM succinate were added to reach maximal coupled respiration, and 10 μM rotenone were injected to obtain the complex II-coupled state 3 respiration. Oligomycin (8 μg/mL) was added to determine the uncoupled state 4 respiration. Finally, FCCP (1 μM) was added to control the permeabilisation of the fibroblasts. All experiments were performed in duplicate on independent cell cultures.

### Statistical analysis

Vehicle-treated and idebenone-treated fibroblasts from LHON patients and controls were compared using a non-parametric Mann-Whitney *U *test; differences were considered significant at *p *< 0.05.

## Results

### Idebenone at 10 μM partially restores complex I enzymatic activity

**Figure 1:**
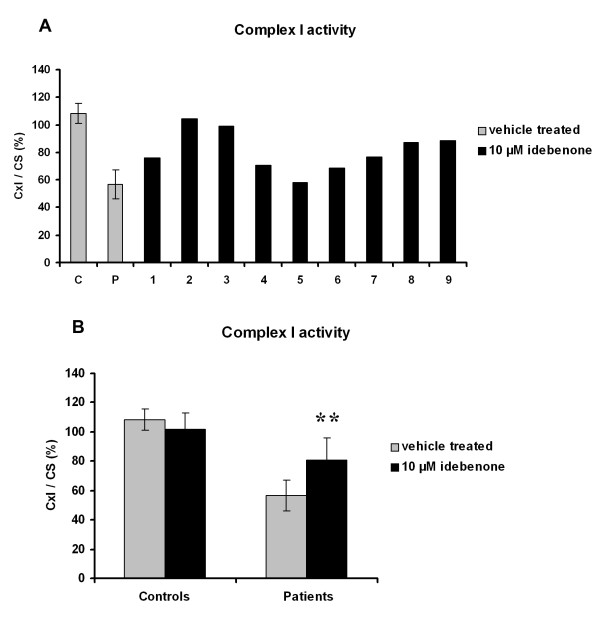
**The effect of idebenone on complex I activity**. **A**. Complex I (CxI) activity in fibroblasts from LHON patients (n = 9) and controls (n = 3) treated with vehicle (*grey bars*) or 10 μM idebenone (*black bars*) 24 h before the analysis. The enzymatic activity was standardized in terms of citrate synthase (CS) activity, indicative of mitochondrial mass and expressed as the percentage of activity of one control fibroblast used as reference in all experiments (CxI/CS(%)). **B**. Pooled results from fibroblasts from LHON patients and controls. Results are expressed as mean values ± SD. Statistical significance: **p < 0.01 compared with vehicle-treated fibroblasts from LHON patients. C = Controls and P = Patients.

The activity of NADH ubiquinone oxidoreductase was lower in fibroblasts from LHON patients as compared to controls (56.7 ± 10.5 in LHON fibroblasts *versus *108.4 ± 7.4 in controls; *p *= 0.01) (Figure 1A). Idebenone at 10 μM induced variable increases in complex I enzymatic activity in fibroblasts from LHON patients, whereas it had no such effect on control fibroblasts (Figure 1A). The pooled results of fibroblasts from LHON patients treated with idebenone showed a 42% increase in complex I enzymatic activity (81.0 ± 15.0 in fibroblasts from LHON patients treated with idebenone *versus *56.7 ± 10.5 in vehicle-treated fibroblasts; *p *= 0.002) (Figure 1B).

### Idebenone at 10 μM induces contradictory effects on respiratory chain activity

**Figure 2:**
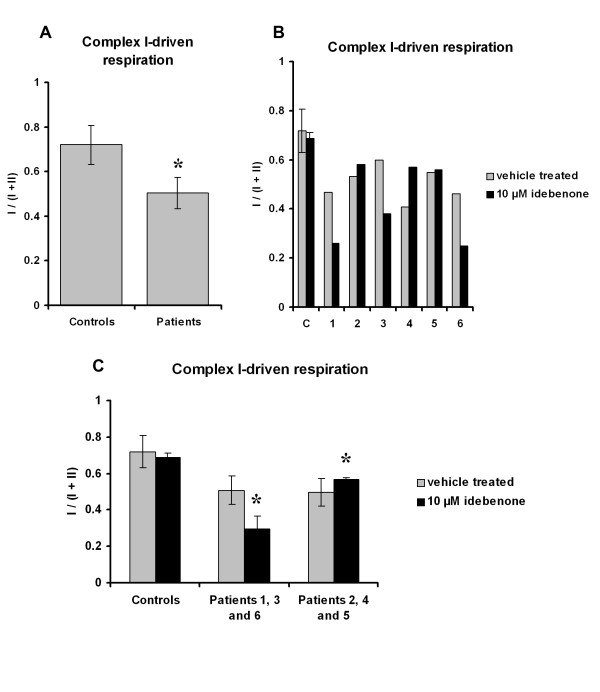
**The effect of idebenone on complex I-driven respiration**. Determination of complex I-driven respiration in permeabilized fibroblasts from LHON patients (n = 6) and controls (n = 3) treated with vehicle (*grey bars*) or 10 μM idebenone (*black bars*). The I/(I+II) ratio corresponds to the fraction of respiration driven by complex I with respect to the maximal respiratory rate driven by complexes I and II. **A**. Pooled results of fibroblasts from Patients 1-6 and controls. **B**. Individual results of Patients 1-6 and controls. **C**. Pooled results of fibroblasts from Patients 1, 3 and 6, in which idebenone impaired respiration, and Patients 2, 4 and 5, in which idebenone stimulated respiration. Results are expressed as mean values ± SD. Statistical significance: *p < 0.05 compared with vehicle-treated fibroblasts from LHON patients. C = Controls and P = Patients.

The activity of the mitochondrial respiratory chain was analysed by polarographic measurements of complex I-driven respiration. The I/(I+II) ratio indicated the fraction of complex I-driven respiration with respect to the maximal respiratory rate controlled by complexes I and II. The I/(I+II) ratio was significantly lower in fibroblasts from six LHON patients compared to controls (0.50 ± 0.05 *versus *0.72 ± 0.08; *p *= 0.02) (Figure 2A). Idebenone had no effect on complex I-driven respiration in controls. However, in fibroblasts from LHON patients the response to idebenone proved to be contradictory. Thus, in fibroblasts from Patients 1, 3 and 6, complex I-driven respiration was impaired, whereas it was stimulated in fibroblasts from Patients 2, 4 and 5 (Figure 2B). The analysis of these two groups of fibroblasts revealed a deleterious effect of idebenone in the first group, with a 42% reduction in complex I-driven respiration compared to controls (*p *= 0.04), and a stimulating effect of idebenone in the second group, with a 16% increase compared to controls (*p *= 0.04) (Figure 2C).

## Discussion and conclusions

Previous studies of LHON fibroblasts have shown significant complex I impairment [3,15]. These results attest that, although the clinical expression is mostly limited to retinal ganglion cells, the OXPHOS defect is probably more generalized as has also been shown by Barbiroli et al. [16] who found defective energy metabolism in the muscle and brain of LHON patients by using 31P-MRS *in vivo*. Thus, fibroblasts represent an interesting model to explore the LHON-associated OXPHOS defect and to research molecules able to compensate the defect.

This study demonstrates the impact of idebenone on respiratory chain activity in fibroblasts from nine LHON patients. Indeed, idebenone increased complex I enzymatic activity in these fibroblasts by 42% compared to controls (*p *= 0.002). This idebenone effect on complex I enzymatic activity was found to be shared by fibroblasts carrying the same m.11778 G > A mutation in 7 different genetic backgrounds (inter-individual variability); this idebenone effect was also found to be shared by fibroblasts carrying the 3 main LHON mutations (inter-mutation variability). The action of idebenone was not due to an increase either of complex I or of the mitochondrial mass. Indeed, two subunits of complex I, i.e. NDUFA9 and NDUFB8 in western blot, and the citrate synthase activity were not affected by the treatment (Additional file 1: Figure S1).

Various types of action have been ascribed to idebenone in the literature. Experiments with idebenone have led to some conflicting results. Thus, the oral administration of idebenone for 3 days has been reported to stimulate mitochondrial respiration linked to complexes I and II in the rat brain [11]. In contrast, idebenone has been described as a weak substrate for complex I activity [17,18]. Idebenone has also been shown to bind to a rotenone-insensitive site, i.e. the non-physiological iron-sulphur N2 site, thus leading to higher NADH oxidation but without complete reduction [19]. However, in these latter observations, idebenone was added in the incubation medium directly only during the experiments. The administration of idebenone over a period of 3 days [11] may have led to better metabolization of the drug, allowing it to enter cells and activate signalling pathways such as MAP kinase [10].

In our study, the polarographic investigation of the effect of idebenone on mitochondrial activity in fibroblasts from six LHON patients led to contradictory results: a 16% increase in complex I-driven respiration in three cases, and a 42% decrease in the other three cases. The increase observed might correspond to the idebenone-induced improvement similar to that reported in intact rat brain mitochondria [11], whereas the opposite response might be explained by an increase of electron escape du to enhanced idebenone fixation at a non-specific site [19].

The variability of the effect of idebenone on complex I activity may be linked to the genetic variability of the LHON patients. However, the number of patients in our study was too small to reveal the possible influence of the haplogroup, the mutation type or the mutation level on the treatment. A larger cohort of LHON patients would be necessary to assess the influence of these factors.

To our knowledge, this is the first demonstration that idebenone directly influences mitochondrial respiratory chain activity in LHON patients. Idebenone increases the enzymatic activity of complex I and acts on the complex I-driven respiration rate, modifying the balance between the electron fluxes provided by complexes I and II. It is tempting to speculate that the variability of the biological response to idebenone in fibroblasts from LHON patients may explain the discordant results observed in some clinical studies [6,7,20]. Our results suggest that only a fraction of LHON patients may benefit from the improved respiration afforded by idebenone. If this finding is substantiated by a more extensive study, a preliminary investigation of the impact of idebenone on fibroblasts from LHON patients may be useful to select potentially "good responders" to the treatment.

## Abbreviations

CS: Citrate synthase; DCPIP: Dichlorophenolindophenol; LHON: Leber's hereditary optic neuropathy; mtDNA: Mitochondrial DNA; ND: NADH dehydrogenase; NUR: NADH ubiquinone reductase; OMIM: Online Mendelian Inheritance in Man; OPA1: Optic atrophy protein 1; OXPHOS: Oxidative phosphorylation.

## Availability of supporting data

The data sets supporting the results of this article are included within the article and its additional file.

## Competing interests

The authors declare that they have no competing interests.

## Authors' contributions

CA conceived and designed the study, performed literature search, conducted experiment, analyzed and interpreted data, wrote and critically revised the manuscript. NG, VDD and AC participated in all stage of study design, data interpretation and critical revision of the manuscript. VG, CV, JC, MF, DM, PAB, DB, VP participated to the clinical and the biological diagnosis of the patients, and critically revised the manuscript. PR and DL conceived and designed the study, provided direction and oversight of the experiments and critically revised the manuscript. All authors have read and approved the final manuscript.

## Supplementary Material

Additional file 1 **Figure S1. The effect of idebenone on complex I quantity and mitochondrial mass**. **A**. Quantity of two subunits of complex I, NDUFA9 and NDUFB8, in fibroblasts from LHON and controls treated with vehicle (V) or 10 μM idebenone (I10) 24 hours before the analysis. **B**. Citrate synthase activity in fibroblasts from LHON patients (n = 9) and controls (n = 3) treated with vehicle (grey bars) or 10 μM idebenone (black bars) 24 hours before the analysis. The enzymatic activity was expressed as the percentage of activity of one control fibroblast used as reference in all experiments. Click here for file
