# Supplementary figures and images for: Idebenone increases mitochondrial complex I activity in fibroblasts from LHON patients while producing contradictory effects on respiration
Source: BMC Res Notes. 2011 Dec 22;4:557. doi: 10.1186/1756-0500-4-557 (PMC3285568; doi:10.1186/1756-0500-4-557)

**A**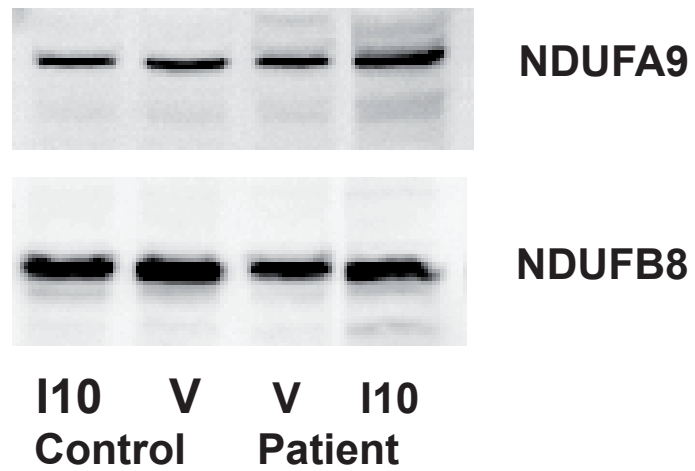**B**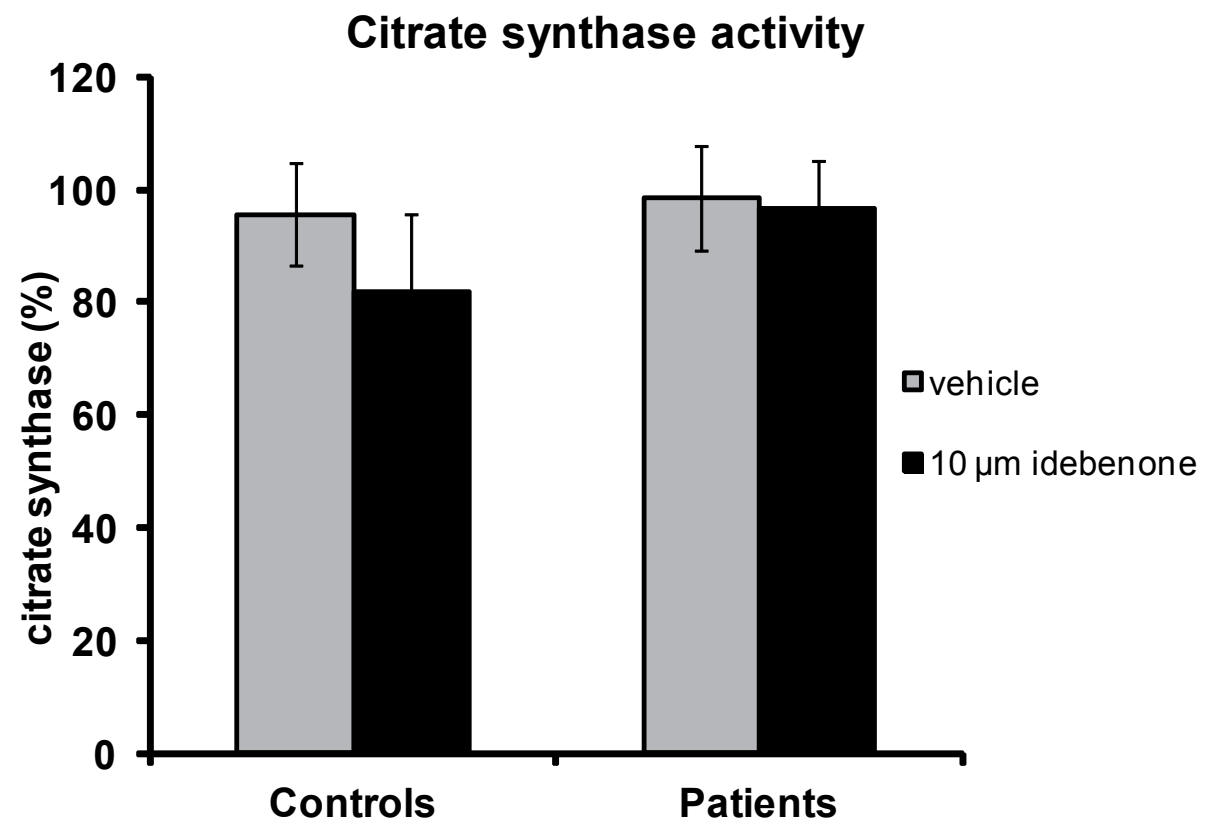

Supplement: Additional file 1 — Figure S1. The effect of idebenone on complex I quantity and mitochondrial mass. A. Quantity of two subunits of complex I, NDUFA9 and NDUFB8, in fibroblasts from LHON and controls treated with vehicle (V) or 10 μM idebenone (I10) 24 hours before the analysis. B. Citrate synthase activity in fibroblasts from LHON patients (n = 9) and controls (n = 3) treated with vehicle (grey bars) or 10 μM idebenone (black bars) 24 hours before the analysis. The enzymatic activity was expressed as the percentage of activity of one control fibroblast used as reference in all experiments. [file 1756-0500-4-557-S1.PDF]
